# Let It Go: Intracardiac Echocardiography Is the Future of Imaging for Left Atrial Appendage Occlusion

**DOI:** 10.1016/j.jscai.2022.100518

**Published:** 2022-11-03

**Authors:** Andrew M. Goldsweig, Mohamad Alkhouli

**Affiliations:** a Division of Cardiovascular Medicine, University of Nebraska Medical Center, Omaha, Nebraska; b Department of Cardiovascular Medicine, Mayo Clinic, Rochester, Minnesota

**Keywords:** ICE, intracardiac echocardiography, left atrial appendage occlusion, transesophageal echocardiography

FACT: No one wants to undergo transesophageal echocardiography (TEE).

FICTION: Left atrial appendage occlusion (LAAO) requires TEE.

Although LAAO requires real-time cardiac imaging, intracardiac echocardiography (ICE) is rapidly emerging as an alternative to TEE. Effective use of ICE in LAAO requires a thorough understanding of its advantages and limitations. Rigorous studies are necessary to evaluate the safety and efficacy of ICE guidance for LAAO. Improved protocols, technology, and reimbursement will streamline ICE use.

## Advantages and limitations of ICE for LAAO

In comparison with TEE, ICE guidance for LAAO offers unique advantages. ICE allows the LAAO operator to control the imaging directly, eliminating the TEE-associated challenge of communication between a procedure’s eyes (the imager) and hands (the procedural operator). Moreover, ICE is minimally invasive. Without prolonged TEE imaging, ICE-guided LAAO can be performed under conscious sedation. In addition to improving patient comfort and satisfaction, ICE improves efficiency and safety by eliminating the time and risks associated with intubation and extubation. This efficiency improves catheterization laboratory throughput and facilitates same-day discharge.^1^ Conscious sedation and same-day discharge became especially important during the COVID-19 pandemic, when both anesthesiologists and hospital beds were scarce.

In contrast to TEE, 2-dimensional ICE provides only single-plane imaging, requires probe manipulation to change views, generates lower far-field image resolution, and necessitates a dedicated venous access site. Although we believe that ICE should be the default imaging modality for LAAO, there remains a minority of cases for which TEE may be appropriate (Table 1), especially for less experienced ICE users.

**Table 1:** Standard intracardiac echocardiography protocol for left atrial appendage occlusion and indications not to use intracardiac echocardiography to guide left atrial appendage occlusion.

ICE protocol for LAAO	

View	Structures seen

Midright atrium anterior view (home view)	Right atrium, tricuspid valve, right ventricle, pulmonic valve, and aortic valve; assess for pericardial effusion
Right side of the interventricular septum	Left ventricle; assess left ventricular function and pericardial effusion
Main pulmonary artery or right ventricular outflow tract	Apex of the left atrial appendage; assess for thrombus
Midright atrium left/posterior view (transseptal view)	Interatrial septum and left atrium; guide transseptal puncture
Retroflexed midleft atrium	Left atrial appendage; similar to 45° TEE view
Proximal left superior pulmonary vein	Left atrial appendage; similar to 90° TEE view
Retroflexed + right-steered supramitral view	Left atrial appendage; similar to 135° TEE view
Anteflexed midleft atrium	Mitral valve and left ventricle; assess for pericardial effusion

Indications not to use ICE guidance for LAAO (when ICE is the default imaging guidance modality)	

Concern for left atrial appendage thrombus (patient not on preprocedural anticoagulation or with a history of thrombus)	
No preprocedural imaging (CTA or TEE)	
A GFR of <30 mL/min per 1.73 m^2^ and not on hemodialysis (ICE-guided procedures may use more contrast than the amount used for TEE-guided procedures)	
Complex anatomy (shallow, very large/small, or posteriorly directed left atrial appendage)	

CTA, computed tomography angiography; GFR, glomerular filtration rate; ICE, intracardiac echocardiography; LAAO, left atrial appendage occlusion; TEE, transesophageal echocardiography.

## Safety and efficacy of ICE for LAAO

Despite its advantages, ICE remains underused for LAAO. More robust data are necessary to confirm the safety and efficacy of ICE guidance for LAAO. The previously published US experience consists of only small single- and dual-center studies^2^; however, in this issue of the *Journal*, Zahid et al^3^ report on 1410 ICE-guided LAAO procedures identified from 61,995 LAAO procedures in the National Inpatient Sample from 2015 to 2019. The use of ICE increased from 1.7% in 2015 to 2.2% in 2019. Patients receiving ICE guidance had more baseline comorbidities than those receiving TEE guidance; however, propensity-matched procedural outcomes were mostly similar, notably including mortality and retroperitoneal bleeding. These data provide the initial multicenter evidence base supporting the safety of ICE-guided LAAO; however, the study has considerable limitations because data from the National Inpatient Sample are not at all granular and the study period preceded the broader use of ICE for LAAO in more recent years.

A larger and more contemporary retrospective data set is expected from the SURPASS analysis of the National Cardiovascular Data Registry LAAO registry, a registry that includes all commercial LAAO procedures in the United States. Ultimately, prospective—preferably randomized—data remain the gold standard, and we hope to see a future randomized controlled trial comparing outcomes between ICE- and TEE-guided LAAO procedures.

## ICE protocols, technology, and reimbursement for LAAO

Furthermore, ICE guidance for LAAO will be bolstered by protocol standardization and improvements in technology and reimbursement. A standardized ICE imaging protocol for LAAO should exclude baseline pericardial effusion and left atrial appendage (LAA) thrombus, guide transseptal puncture, measure the LAA at multiple angles, assess the occlusion device at multiple angles, and exclude postprocedural pericardial effusion. Our standard protocol is provided in Table 1. A 2021 expert consensus document further describes how to obtain optimal ICE views of the LAA.^4^

The advent of 3-dimensional (3D)-ICE further expands the capabilities of ICE guidance for LAAO.^5^ Although 3D images are visually appealing, the key innovation with 3D-ICE is the ability to rotate the imaging plane digitally, similar to TEE, to obtain a full 360° visualization of the LAA. This digital rotation capability permits numerous LAA measurements and thorough inspection of the implanted device before release without the need for excessive maneuvering of the probe in the left atrium.

In 2022, the American Medical Association’s Current Procedural Terminology Panel and the Center for Medicare and Medicaid Services added LAAO (33340) to the list of primary procedures with which the ICE add-on Current Procedural Terminology code (+93662) may be billed; however, reimbursement for intraprocedural ICE remains significantly lower than reimbursement for TEE. Furthermore, only the LAAO operator may bill the add-on code. For ICE imaging to become the routine standard of care for LAAO, reimbursement must catch up. In particular, stand-alone reimbursement must be made available to cover the services of an imaging physician or technologist to manage the ICE imaging console, especially for controlling the more labor-intensive 3D-ICE interface.

In conclusion, ICE offers important advantages for LAAO procedural guidance. Rigorous studies have begun to demonstrate the safety and efficacy of ICE to guide LAAO. Evolving protocols, technology, and reimbursement will help to systematize the use of ICE. Given these many merits of ICE, the LAAO community should embrace ICE as the ascendant standard of imaging for procedural guidance. In the coming years, the use of ICE will undoubtedly continue to increase rapidly; as Elsa (Idina Menzel) sings in Disney’s *Frozen*, “Let it go, let it go, can’t hold it back anymore!”
